# Digital solutions to optimize guideline-directed medical therapy prescription rates in patients with heart failure: a clinical consensus statement from the ESC Working Group on e-Cardiology, the Heart Failure Association of the European Society of Cardiology, the Association of Cardiovascular Nursing & Allied Professions of the European Society of Cardiology, the ESC Digital Health Committee, the ESC Council of Cardio-Oncology, and the ESC Patient Forum

**DOI:** 10.1093/ehjdh/ztae064

**Published:** 2024-08-30

**Authors:** Mark Johan Schuuring, Roderick Willem Treskes, Teresa Castiello, Magnus Thorsten Jensen, Ruben Casado-Arroyo, Lis Neubeck, Alexander R Lyon, Nurgul Keser, Marcin Rucinski, Maria Marketou, Ekaterini Lambrinou, Maurizio Volterrani, Loreena Hill

**Affiliations:** Department of Biomedical Signals and Systems, University of Twente, Drienerlolaan 5, 7522 NB Enschede, The Netherlands; Department of Cardiology, Medical Spectrum Twente, 7512 KZ Enschede, The Netherlands; Department of Cardiology, Leiden University Medical Centre, Leiden, The Netherlands; Department of Cardiovascular Imaging, King’s College London, Croydon Health Service London, London, UK; Steno Diabetes Centre Copenhagen, 2730 Herlev, Denmark; Department of Cardiology, Erasme Hospital, Université Libre de Bruxelles, Brussels, Belgium; Centre for Cardiovascular Health, Edinburgh Napier University, Edinburgh, UK; Cardio-Oncology Service, Royal Brompton Hospital, Guys and St. Thomas NHS Foundation Trust, London, UK; Faculty of Medicine, Department of Cardiology-Istanbul, Istanbul Health Sciences University, Istanbul, Turkey; Poland, ESC Patient Forum, European Society of Cardiology, Sophia Antipolis Cedex, France; Cardiology Department, Heraklion University Hospital, Stavrakia, Heraklion, Greece; Department of Nursing, Cyprus University of Technology, Limassol, Cyprus; Cardiology Rehabilitation Unit, IRCCS San Raffaele, 00163 Rome, Italy; School of Nursing and Midwifery, Queen’s University Belfast, Belfast, UK

**Keywords:** Digital health, Heart failure, GDMT, Up-titration, Remote contact, Pharmacotherapy

## Abstract

The 2021 European Society of Cardiology guideline on diagnosis and treatment of acute and chronic heart failure (HF) and the 2023 Focused Update include recommendations on the pharmacotherapy for patients with New York Heart Association (NYHA) class II–IV HF with reduced ejection fraction. However, multinational data from the EVOLUTION HF study found substantial prescribing inertia of guideline-directed medical therapy (GDMT) in clinical practice. The cause was multifactorial and included limitations in organizational resources. Digital solutions like digital consultation, digital remote monitoring, digital interrogation of cardiac implantable electronic devices, clinical decision support systems, and multifaceted interventions are increasingly available worldwide. The objectives of this Clinical Consensus Statement are to provide (i) examples of digital solutions that can aid the optimization of prescription of GDMT, (ii) evidence-based insights on the optimization of prescription of GDMT using digital solutions, (iii) current evidence gaps and implementation barriers that limit the adoption of digital solutions in clinical practice, and (iv) critically discuss strategies to achieve equality of access, with reference to patient subgroups. Embracing digital solutions through the use of digital consults and digital remote monitoring will future-proof, for example alerts to clinicians, informing them of patients on suboptimal GDMT. Researchers should consider employing multifaceted digital solutions to optimize effectiveness and use study designs that fit the unique sociotechnical aspects of digital solutions. Artificial intelligence solutions can handle larger data sets and relieve medical professionals’ workloads, but as the data on the use of artificial intelligence in HF are limited, further investigation is warranted.

## Graphical Abstract

**Graphical Abstract:**
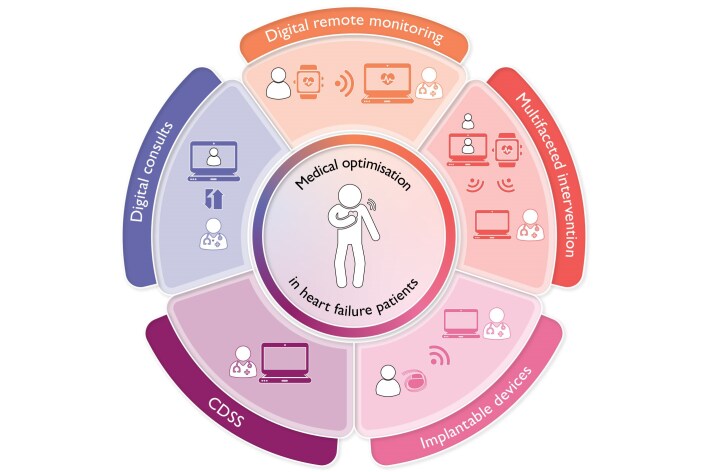
CDSS, clinical decision support system.

## Background

Heart failure (HF) affects >64 million people worldwide, and it is estimated that >10% of the population above 70 years old is affected by HF.^1–4^ Recent data suggest plateaus or reversals after long-standing declines in cardiovascular mortality, particularly for HF-related mortality.^5^ Current treatment focuses on symptom management and treating the underlying cause of HF, as well as optimizing the heart rate, blood pressure, and haematologic parameters such as haemoglobin and ferritin by prescribing guideline-directed medical therapy (GDMT).^1,2,6^ The primary objectives of prescribing GDMT in HF care are to alleviate the cardiac workload and enhance contractility. Neurohormonal activation plays a critical role in the pathophysiology of HF, triggering a cascade of adverse effects, including myocardial remodelling. This process involves the structural and functional alteration of the heart muscle, typically characterized by ventricular dilatation, hypertrophy, and fibrosis, which ultimately compromise cardiac output and efficiency. Guideline-directed medical therapy targets key neurohormonal pathways to mitigate these detrimental changes. For instance, beta-blockers and angiotensin-converting enzyme (ACE)-inhibitors are utilized to counteract the effects of excessive sympathetic stimulation and renin–angiotensin–aldosterone system activation. By doing so, they help to stabilize and sometimes reverse adverse myocardial remodelling.^7^ Optimal achievement of GDMT for HF results in improvement of patient’s symptoms, quality of life, and overall prognosis.^8–18,19^ De-escalation/discontinuation of GDMT prescriptions has been shown to increase the risk for HF hospitalization and all-cause mortality.^20,21^ The combination of pharmacotherapy classes has been proven to have a synergistic effect, providing greater benefits compared to using a single pharmacotherapy class alone.^22^ In a systematic review and network meta-analysis including 75 relevant trials representing 95 444 participants, a combination of four recommended pharmacotherapy classes was most effective in reducing all-cause death (hazard ratio: 0.39; 95% confidence interval: 0.31–0.49).

Guideline-directed medical therapy with an indication in the European Society of Cardiology (ESC) guidelines for patients with HF with reduced ejection fraction (HFrEF) include a sodium-glucose transporter inhibitor (SGLT2i), an angiotensin receptor/neprilysin inhibitor (ARNI), an ACE inhibitor, a mineralocorticoid receptor antagonist (MRA), and beta-blockers.^1^ The 2023 Focused Update on the HF guideline also recommends a SGLT2i for HF with preserved ejection fraction.^2^

## Optimization of guideline-directed medical therapy

Optimization of GDMT prescription rates refers to pharmacotherapy initiation and dose adjustments to achieve a comprehensive evidence-based regimen.^1,2,23,24,25^ Optimal prescription rates are often not reached when pharmacotherapies are not titrated to recommended levels, adjustments to GDMT have not been made over time, symptoms persist, and/or patients have a worsening clinical status. Effective strategies to optimize prescription of GDMT are essential in the overall treatment of patients with HF.^26,27^ The 2023 Focused Update on the HF guideline has a recommendation of rapid optimization of GDMT before discharge and during the first 6 weeks following a HF hospitalization.^2^ Various optimization algorithms have been described on the sequencing of prescribing GDMT.^23,28–31^ In-hospital optimization of GDMT prescription rates has reliably demonstrated substantial improvements in post-discharge GDMT prescription rates, as compared with deferring optimization to the outpatient setting.^32–34^ The responsibility for optimizing GDMT in HF care increasingly involves nurse-led strategies, with some nurses taking on advanced roles, including the prescription and adjustment of medications. While such advanced practice roles are well established in parts of Europe, there is significant variability in their availability globally.^35^ This disparity is due to differences in healthcare infrastructure, training, and regulatory frameworks.

However, in clinical practice, there remains a substantial proportion of prescribing inertia, time lag in optimization, and low target dose achievement of GDMT.^36–43^ Data from the EVOLUTION HF study including 266 589 patients demonstrated that mean times from the patient hospitalized with HF to optimization of GDMT prescription rates were longer for novel GDMTs (SGLT2i and ARNI) than for other GDMTs like MRA and ACE-inhibitor: 39 and 44 vs. 12 and 13 days (Japan), 44 and 33 vs. 22 and 31 days (Sweden), and 33 and 19 vs. 18 and 24 days (USA).^36^ The explanation for worldwide underuse of GDMT is historical and multifactorial but includes limitations in resources and diverse prescribing practices across clinicians and healthcare organizations.^44–46^ Especially in the case of a decline in personnel, logistical challenges arise, necessitating the simultaneous implementation and innovation of strategies to reduce risks for patients with HF.^47^ Other significant barriers of GDMT optimization are the lack of awareness and education among medical clinicians, and the limited time spent with patients to ensure that they have sufficient health literacy to adhere to their prescribed pharmacotherapy. Comorbidities are sometimes mentioned as a contraindication for GDMT optimization; however, the STRONG-HF trial did not support this claim.^48^ Some clinicians are hesitant to prescribe optimal GDMT for patients due to concerns about side effects, cost, or simply the inconvenience for patients of managing a complex pharmacotherapy regimen.^49^ Cultural and innate beliefs about pharmacotherapy can greatly influence the decision to take medications as prescribed.^50^ Furthermore, patients with HF who are on non-GDMT polypharmacy have a lower chance of achieving optimal GDMT at follow-up.^51^

## Improvement of optimization of guideline-directed medical therapy

To address the challenge of GDMT prescription inertia in patients with HF, fostering a behavioural shift among clinicians is essential. By promoting a proactive and guideline-driven mindset, clinicians can optimize GDMT better, improving patient outcomes. Digital solutions can support clinicians in this strategy to achieve better optimization of GDMT prescription rates in various ways.^52,53–55^ Digital consults for instance not only save valuable travel time for patients but also enable clinicians to efficiently see more patients with HF within the same time span, enhancing their capacity to prescribe GDMT and improving overall patient care. Digital remote monitoring offers valuable insights and additional data for clinicians through frequent home blood pressure and heart rate measurements, fortifying the optimization of GDMT to ensure a more robust and safe treatment approach.^56,57^ A clinical decision support system (CDSS) can serve as a valuable ally, offering clinicians essential knowledge and timely alerts, enabling a proactive approach to GDMT prescription for patients with HF. Digital solutions are also effective when integrated synergistically, not as standalone entities, forming multifaceted interventions such as remote monitoring as well as digital feedback to clinicians that harness their combined strength for optimal impact.^58–61^ Digital solutions gained popularity during the COVID-19 pandemic due to their ability to address access challenges by providing remote and convenient access to healthcare services and information, ensuring continuity of care despite physical constraints.^62,63^ The increased popularity of digital solutions during and following the pandemic has accelerated lessons learned, driving continuous improvement and refinement of these technologies to better meet evolving healthcare needs.^58,64–71^ The Clinical Consensus Statement provides clinicians and researchers with examples of digital solutions, gives insight on clinical implementation, highlights potential barriers to adoption and lists strategies to ensure equality of care.

## Digital consults

### Key features

Digital consults redefine healthcare by enabling remote accessibility and convenience^52,72^ (see *Table 1*). Patients can connect with healthcare providers from any location, therefore eliminating the need for their physical presence. This is facilitated through multimodal communication channels, including video and audio calls, and text messaging, providing flexibility in interaction. Asynchronous communication allows patients to engage with healthcare providers at their own pace. Messaging platforms enable patients to share updates or seek guidance outside of scheduled appointments, which can contribute to a patient-centred, technology-driven healthcare experience.

**Table 1:** Key features, best practices, and gaps in knowledge

		Development/research		Implementation	
	Key features	Best practices	Gaps in knowledge	Best practices	Gaps in knowledge
Digital consults	Remote accessibility and convenience	Integration of electronic health records	Optimal mode unknown	Security and privacy measures	Handle digital literacy
	Multimodal communication channels	Use secure and reliable platforms		Standardization practice	Long-term effect on patient–clinician relationship
	Asynchronous communication	Interoperability of the devices		Interoperability of the devices	
Digital remote monitoring	Real-time data collection	Integration of EHR with wearables and monitoring devices	Optimal timing of intervention in deteriorating patients	Patient engagement tools	Optimal cost and resource allocation
	Remote accessibility	Customizable alerts and notifications	Automated (AI) handling of data	Scalable solutions	Optimal set of parameters to monitor
	Data analytics and trend analysis			Reliable Wi-Fi and network connections	
Digital monitoring through implantable devices	Sensors for continuous monitoring	Remote programming and updates	Optimal effect on battery life	Customizable alerts	Achieve integration with electronic health records with various vendors
	Newer models with remote data transmission	Multiparameter monitoring	Miniaturization limits		
Digital clinical decision support systems	Computer-based tool	Evidence-based recommendations	Extend of personalized decision support needed	Patient and clinician training and education	Optimal evaluation of clinical impact
	Knowledge base	Integration with electronic health records	Optimal evaluation of clinician experience	Data security and privacy compliance	Optimal workflow integration
	Alerts and reminders	Customization for workflow integration	Long-term impact on patient outcome		

EHR, electronic health record.

### Interpretation of the literature

Virtual peer-to-peer consultation is an example of a digital consultation used to evaluate the efficacy to optimize GDMT prescription rate for HF patients (see *Table 2*).^73^ Patients admitted to a non-cardiology ward were included, and blood pressure and laboratory results were systematically evaluated. A significant increase in the proportion of patients on GDMT was reported, but the sample size was small (*n* = 91). Results are comparable to the IMPLEMENT-HF pilot randomized controlled trial (RCT), where a pharmacist–clinician team provided evidence-based pharmacotherapy recommendations in a non-cardiology setting.^74,75^ The digital consultation demonstrated safety and significantly impacted GDMT optimization during patients’ time in hospital. In a post-discharge outreach study that used a videophone application to support nurse–patient communication after hospital discharge,^76^ compared to usual care and telephone intervention, videophone intervention showed increased medication adjustments over the short follow-up period (3 months). This synthesis of recent research highlights promising interventions to enhance GDMT in HF patients, spanning in-hospital consults and post-discharge outreach.

**Table 2:** Current literature on digital solutions to optimize guideline-directed medical therapy in heart failure patients

First author (acronym)	Year	Design	Sites	Population	*n* (total)	*n* (int)	FU (m)	Effect of intervention
Digital consultation								
Bhatt (IMPLEMENT-HF)	2021	Prospective cohort	1	Hospitalized HFrEF	118	89	1	Increase in beta-blocker, ARNI, MRA, and triple therapy
Bhatt	2023	Prospective cohort	3	Hospitalized HFrEF	252	83	6	Increase GDMT optimization score
Rao	2023	RCT	1	Non-cardiology ward patients	91	52	1	Increase proportion of patients on ACE/ARB/ARNI
Sammour	2022	Retrospective cohort	?	HFrEF	5439	2610	?	MRA and SGLT2i more often started than telephone contact
Yuan	2021	Retrospective cohort	31	Ambulatory HF during COVID	10 591	1009	9	Lower beta-blocker, ACE-i/ARB/ARNI, and MRA as compared to in-person visit
Wakefield	2009	RCT	1	Hospitalized HF	148	52	3	More likely medication adjustments
Digital remote monitoring								
Antonicelli	2008	RCT	1	Congestive HFrEF	57	28	12	Increase beta-blocker use
Artanian	2020	RCT	1	Stable HFrEF	42	21	6	Higher proportion of optimal GDMT doses, decreased time to dose optimization
Brahmbhatt	2022	RCT	5	HF	108	56	24	Increase number of patients achieving maximum dose, and earlier
Dierckx	2015	Retrospective cohort	?	HFrEF	333	278	6	Similar beta-blocker, ACE-I/ARB, and MRA use
Giordano	2011	Retrospective cohort	1	Chronic HFrEF	358	238	6	Increase in beta-blocker
Koehler (TIM-HF2)	2018	RCT	113	LVEF <45%, NYHA II/III	1571	796	12	3546 medication (likely GDMT) changes
McLachlan	2021	Prospective cohort	1	Acute HF and HFrEF	50	50	2	Increase use renin–angiotensin blocker, beta-blocker, spironolactone, and ARNI
Romero	2023	RCT		HFrEF	55		6	Increase GDMT optimization score
Samsky (VITAL-HF)	2023	Prospective cohort	1	HFrEF	12	12	3	10 initiations, 52 up- and 13 down-titrations GDMT
Wong (DAVID-HF)	2022	Prospective cohort	1	HFrEF	20	20	4	Increase GDMT target dose
Digital monitoring through implantable devices								
Abraham (CHAMPION)	2011	RCT	64	NYHA III, previous HF admission	550	245	6	More optimization ACE-i, beta-blocker, and loop diuretic
Bohm (OptiLink HF)	2016	RCT	65	HFrEF and ICD implant	1002	505	23	0.37 GDMT changes per 6 months
Brugts (MONITOR-HF)	2023	RCT	25	Chronic HF NYHA III	348	176	48	Individualized modification of GDMT
D'Onofrio	2015	*Post hoc*	25	ICD/CRTD patients	987	499	12	No association RM and beta-blocker use
Hernandez (MANAGE-HF)	2022	Prospective cohort	29	HFrEF and CIED	200	200	12	GDMTs were increased during 74% of the alert cases
van Veldhuisen (DOT-HF)	2011	RCT	72	Chronic HF, ICD implant	335	168	15	Diuretics change more in access arm than control arm
Digital support in electronic health records								
Ahmad (REVEAL-HF)	2022	RCT	4	Hospitalized HF	3124	1590	12	No effect on GDMT prescription rate
Allen (EPIC-HF)	2021	RCT	6	HFrEF	290	145	1	Intensification of GDMT
Brahmbhatt (Medly Titrate)	2024	RCT	1	HFrEF	108	56	6	Effective, safe, feasible GDMT optimization
Ghazi (PROMPT-HF)	2022	Cluster RCT	4	Ambulatory HFrEF	1310	1310	1	Higher rates of GDMT
Ghazi (PROMPT-AHF)	2023	Cluster RCT	4	Hospitalized HF	1012	1012	^a^	No higher rates of GDMT
McCarren	2013	Cluster RCT	12	All HF patients	220	220	6	Increase in beta-blocker
Mukhopadhyay (BETTER CARE-HF)	2023	Cluster RCT	?	HFrEF	2211	755	1	Increased MRA use
Verma (DASH-HF)	2023	RCT	1	HFrEF	300	150	3	No improvement GDMT optimization score
Combination of digital interventions								
Gulizia (BLITZ-HF)	2022	Cross-sectional	106	Acute and chronic HF	7218	7218	3	Ambulatory HFrEF patients increase in ARNI
Lynch	2022	Prospective cohort	1	Any HF	38	38	12	Increased number and dose GDMT
Rahimi (SUPPORT-HF2)	2020	RCT	7	HFrEF	202	101	6	No improvement GDMT
Slade	2022	Prospective cohort	1	HFrEF	12	12	14	ACE-I/ARB/ARNI/beta-blocker at ≥50% target doses increased

CDSS, clinical decision support system; CIED, cardiac implantable electronic device; EHR, electronic health record; GDMT, guideline-directed medical therapy; HF, heart failure; HFrEF, heart failure with reduced ejection fraction; *n*, number; m, months; RCT, randomized controlled trial; ARNI, angiotensin receptor/neprilysin inhibitor; MRA, mineralocorticoid receptor antagonist; ?, unknown.

^a^Variable (discharge).

### Best practices and gaps in knowledge

Best practices in the development and research of digital consults involve integrating electronic health records (EHRs) to ensure a more seamless data flow.^77^ Utilizing secure and reliable platforms is crucial for safeguarding patient information. It also involves striving for standardization to enhance uniformity, interoperability, and a consistent experience for patients and clinicians.^78^ However, there is also a recognized need for customizability and flexibility in clinical encounters, particularly in situations where individualized care or unique patient needs are paramount. This approach enhances efficiency, privacy, and reliability, fostering the successful adoption and sustained effectiveness of digital consults in healthcare settings. Best practices for digital consults to optimize GDMT in patients with HF focus on structured and concise information delivery to prevent overload and reduce clinician burnout. Ensuring regular training and support for clinicians can help maintain their clinical skills and competence.^79^ However, gaps in knowledge persist regarding the long-term impact of digital consults on skill atrophy, necessitating further research to develop comprehensive strategies. Gaps in knowledge regarding digital consults also include uncertainty about the optimal mode—whether video, audio, or text-based interactions yield the best outcomes for patients with HF. Expanding digital consults in healthcare necessitates widespread access to smartphones, tablets, and reliable Wi-Fi to ensure all patients with HF can benefit from these digital services.^79^ Addressing digital literacy challenges is crucial, as not all patients with HF may be adept at navigating digital healthcare.^80^ Additionally, understanding the long-term effects on the patient–clinician relationship remains elusive. Identifying strategies to maintain the personal connection and trust between the clinician and patient within a virtual setting is essential to ensure the sustained success and acceptance of digital consults in the evolving landscape of healthcare.^81^ Best practices for interoperability of systems given the numerous EHR vendors include adopting universal standards and ensuring seamless data exchange between platforms to enhance care coordination. However, gaps in knowledge exist regarding the implementation and scalability of these standards across diverse healthcare settings, highlighting the need for further research and development. Best practices for the regulatory implications of digital consults include clear guidelines on liability in case of errors, but gaps in knowledge remain regarding the precise delineation of responsibility among clinicians and vendors.

## Digital remote monitoring

### Key features

Digital remote monitoring involves the continuous collection of real-time data from various sources to track and analyse health metrics. This process offers remote accessibility, allowing clinicians to monitor patients’ health from anywhere. Data analytics and trend analysis play a pivotal role, transforming raw information into actionable insights. Through (real-time) data collection, remote accessibility, and sophisticated analytics, digital remote monitoring provides a comprehensive approach to healthcare management, empowering both patients and clinicians with timely and informed decision-making.^82^

### Interpretation of the literature

Digital solutions in patients with HF that evaluate digital remote monitoring have been primarily investigated in pilot RCTs (trials with fewer than 100 HF patients),^83–87^ with one clinical trial in a large academic medical centre performed on this subject.^88^ A such, conclusions on this subject should be drawn with caution. A limiting factor was the heterogeneous design of the digital solution, follow-up, and protocol of optimization of a new pharmacotherapy or dose adjustments. Generally, most pilot studies that reported a positive effect on time to dose optimization, increased use of GDMT, or reduction in visits were studies that combined blood pressure monitoring with regular digital visits. These studies involved a nurse supervised by a cardiologist, in which GDMT was adjusted according to the patient’s blood pressure or a combination of blood pressure, weight, and symptoms.^83–87^ In some studies, a working collaboration between pharmacists, nurses, and clinicians was formed, with pharmacists and nurses optimizing GDMT doses and cardiologists initiating new pharmacotherapy. These studies showed promising results with an increase in prescribing of GDMT.^85,89^ Frequency of contacting patients varied, but most studies report evaluating data at least once a week.^83–87,90–93^ Apart from the pilot studies, one major RCT evaluated the effect of telemonitoring on readmissions and cardiovascular mortality. This trial, the Telemedical Interventional Management in HF II (TIM-HF2) trial, included patients admitted due to acute HF within 12 months before randomization, consequently randomizing them to either telemonitoring or usual care.^86^ Telemonitoring consisted of a combination of devices that allowed the patient to transfer two-channel electrocardiograms (ECGs), blood pressure, weight, and peripheral oxygen saturation to the trial site. The study reported 3546 GDMT changes in the telemonitoring group.^94^ Despite the trial showing very promising results, it remains to be investigated whether these outcomes can be corroborated in patients without 24/7 h surveillance. A real-world setting may show very different results,^93,95^ reinforcing that the use of digital solutions is an active process.^96^ One RCT studied 55 patients with HFrEF who were randomly assigned to receive either usual care or a quality improvement remote dose titration with telemonitoring intervention.^97^ The intervention group used wireless devices to transmit heart rate, blood pressure, and weight data daily, which were remotely reviewed by cardiologists and nurses every 2–4 weeks. At the 6-month follow-up, the intervention group had a GDMT score of 64.6% compared to 56.5% in the usual care group (*P* = 0.01).^91^ These promising results indicate the value of digital remote monitoring through collaborative multidisciplinary approach. At an experimental stage, key examples that push the boundaries of remote monitoring include voice-based speech analysis systems and seismocardiography.^98,99^ However, these are tailored towards volume status assessment rather than GDMT optimization.

### Best practices and gaps in knowledge

In the realm of digital remote monitoring, best practices involve integrating EHR and monitoring devices during clinical implementation, ensuring seamless data flow. Customizable alerts and notifications will enhance user engagement by requesting from the patient only relevant information. Wearables might facilitate GDMT optimization in patients with HF by monitoring biometric signals like heart rate, ECG, and activity levels. Secure transmission to clinicians is required for adequate data handling. However, gaps in knowledge remain on dedicated outcome studies, long-term reliability, and integration into clinical workflows. During implementation, incorporating patient engagement tools such as a quiz or a game fosters active participation. Scalable solutions support flexibility and accommodate growing demands, ensuring the sustained effectiveness of digital remote monitoring systems in delivering personalized and efficient healthcare solutions. Gaps in digital remote monitoring research and development include uncertainties about the optimal timing of interventions for patients with deteriorating HF symptoms and the role of automated [artificial intelligence (AI)] data handling. Relevant studies are ongoing, such as the VITAL-HF (NCT05602454) evaluating virtual care delivery via a specialized platform, with data transmitted to the treating clinician who will create care plans for pharmacotherapy optimization and make clinical decisions.^100^ Some of these AI-guided tools focus on the early detection of decompensation to support GDMT optimization in patients with HF.^101^ However, clinical outcome studies such as the AIM-POWER study (BiovitalsHF solution, NCT04191330) are still being performed. Implementation challenges involve determining the optimal cost and resource allocation for sustained system effectiveness. Defining the optimal set of parameters to monitor remains a key knowledge gap, influencing the precision and efficiency of digital remote monitoring systems in enhancing patient outcomes and healthcare resource utilization.

## Digital remote monitoring through cardiac implantable electronic devices

### Key features

The spectrum of cardiac implantable electronic devices (CIEDs) is increasing with implantable loop recorders, pulmonary artery pressure (PAP) monitors, pacemakers, or defibrillators.^102^ Cardiac implantable electronic device incorporates sensors for continuous monitoring and provides real-time data on cardiac activity. Secure data transmission ensures the confidentiality and integrity of transmitted health information. Newer CIED models feature remote data transmission capabilities, allowing healthcare providers to access patient data remotely. These technological advancements enhance the patient care pathway to optimize GDMT prescriptions.^82,103^

### Interpretation of the literature

The MANAGE-HF cohort study included a total of 200 patients with HFrEF, NYHA class II/III, who received a cardiac resynchronization therapy-defibrillator, or ICD in combination with remote data monitoring. Inclusion criteria included either a hospitalization for HF or unscheduled visit for HF exacerbation or an elevated natriuretic peptide.^104^ Heart failure treatment, including GDMT, was optimized during 74% of the 585 alert cases and during 54% of the 3290 weekly alerts. The OptiLink HF study included 1002 chronic HF patients with an ICD and assessed pulmonary congestion via telemedicine with a defined intervention algorithm. On average, patients in the intervention arm had 0.37 GDMT changes per 6 months as a result of digital remote monitoring.^105^ In the CHAMPION study where PAP monitor pressures were remotely made available to investigators and found that GDMT was changed more often in the remote group using pressure information compared with the control group using symptoms and daily weights.^106^ In the MONITOR-HF study, a RCT, a significant proportion of optimization of GDMT was found during 48 months of follow-up.^7^ These studies indicate the feasibility of using such data in order to improve GDMT optimization. The DOT-HF study on the OptiVol algorithm included 335 chronic HF patients with an ICD and studied digital remote monitoring with intrathoracic impedance measurements.^107^ The trial was terminated as a result of slow enrolment, and only a retrospective chart review was reported on changes of diuretic dose. The investigators reported more diuretic medication changes in the intervention arm (46%) than in the control arm (31%, *P* = 0.041). In contrast, a neutral effect on GDMT prescriptions in HFrEF patients with a CIED being remotely monitored has also been reported.^108^

### Best practices and gaps in knowledge

In the research and development of CIED, best practices include integrating remote programming and updates, facilitating efficient adjustments without physical interventions. Multiparameter monitoring enhances device capabilities by collecting comprehensive health data that could be used by the clinician. Incorporating customizable alerts ensures a patient-centric approach, allowing tailored notifications based on individual health parameters. These practices collectively contribute to the effectiveness, adaptability, and personalized functionality of CIEDs, promoting optimal patient care in cardiac health management. Gaps in CIED development include uncertainties about optimizing battery life, due to the balance between device longevity and patient safety. Miniaturization limits pose challenges, requiring careful consideration of size reduction and possible impact on functionality. Implementation challenges involve achieving seamless integration and updates with EHR from various vendors, necessitating standardized protocols to enhance interoperability and ensure effective data exchange for comprehensive patient care in a diverse healthcare ecosystem.

## Clinical decision support systems

### Key features

At the point of care, CDSS empowers clinicians with evidence-based recommendations to guide care. Imagine, for instance, a smart CDSS that prompts clinicians with warnings and suggestions for potential medication interactions during pharmacotherapy selection, directly within their workflow based on the patient’s medical history. This real-time guidance can help prevent harmful drug interactions and ensure optimal care. Clinical decision support system can help clinicians to ensure that their patients with HF are receiving GDMT in accordance with current guidelines, particularly if integrated in the ESC Pocket Guidelines App or EHR. Clinical decision support system can help with automated alerts and notifications for abnormal or critical laboratory values. These alerts prompt clinicians to take timely actions, encouraging appropriate interventions when needed.

### Interpretation of the literature

The Medly RCT reported that digital optimization of GDMT in 108 patients with HFrEF was effective, safe, feasible, and increased the proportion of patients achieving target doses, in a shorter period of time with no excess adverse events compared with usual care.^109^ A prospective study on CDSS alerts that provided specific guidance on the medical management of patients with chronic HF resulted in an increase in GDMT prescription rates compared to no alerts.^110^ However, the strategy proved unsuccessful in a study with patients with acute HF; therefore, further refinement and improvement of such alerts and changes to clinician incentives are needed.^111^ The BETTER CARE-HF study examined two automated, EHR-embedded tools vs. usual care on MRA prescribing in eligible patients with HFrEF.^112^ Alerts increased MRA prescribing, compared to both a message and usual care. However, differing results were found in the REVEAL-HF study, which examined the impact of including information on patients’ 1 year mortality within EHR on clinical decision-making (i.e. prescription of GDMT) and patient outcomes. The REVEAL-HF study concluded that there was no effect on clinical decision-making.^113^ Use of standardized order sets within EHR can improve GDMT prescription rates by clinicians.^114,115^ Such is particularly relevant when acknowledging the disparities in the GDMT prescription rate between patients admitted as an inpatient to a general ward compared to those admitted to a cardiology ward.^116^ The DASH-HF was a RCT that recruited 300 veterans with HFrEF.^117^ The intervention was a HF dashboard in EHR to monitor and improve outpatient HF management. The study found no significant difference between the intervention arm and usual care arm in GDMT prescription rate. Another pharmacist-led HF medication titration clinic study used a standardized titration protocol that included a patient dashboard.^118^ During a 14-month follow-up period, the prescribing of ACE-I/ARB/ARNI and beta blocker therapy at ≥50% target doses for patients with HFrEF was increased. This study demonstrated the value of a multifaceted, team-based approach that integrates population-level interventions such as clinical dashboard management with a pharmacist-led HF medication optimization clinic.

### Best practices and gaps in knowledge

Best practices for a CDSS development include integration with EHR to enhance data accessibility and continuity of care. During implementation, prioritizing patient and clinician training promotes effective system utilization. These practices collectively contribute to the successful development and implementation of CDSS, improving healthcare outcomes and clinician satisfaction.^113–118^ Gaps in CDSS development include uncertainty about the extent of personalized decision support required, necessitating a nuanced understanding of clinician needs. Optimally evaluating clinician experience remains a challenge, requiring refined assessment methodologies. Understanding the long-term impact of CDSS on patient with HF outcomes is still evolving. During implementation, achieving optimal workflow integration is a knowledge gap, as seamless incorporation into existing organizational processes is crucial to maximize the effectiveness and acceptance of CDSS in healthcare settings.

## Multifaceted interventions

### Key features

Several digital and non-digital interventions can be combined to a multifaceted approach.^57^ A multifaceted approach offers comprehensive benefits for treating HF by addressing its diverse aspects. This holistic and tailored strategy enhances treatment effectiveness.

### Interpretation of the literature

The effect of patient activation with a 3-min video and a one-page digital checklist were evaluated prospectively in the EPIC-HF study.^110^ Guideline-directed medical therapy prescription rate intensified significantly in the intervention group, as compared to the usual care group.^119^ Blitz-HF was a multicentre cross-sectional study that evaluated clinicians’ adherence to HF guidelines and assessed the effect of a web-based system with pop-up reminders on guideline recommendations alongside expert educational meetings. Patients (*n* = 7218) were recruited from ambulatory units or those recently admitted to hospital with HF. Results found that for patients with chronic HF, the prescription for ARNI doubled (*P* = 0.0001); however, combination of GDMT prescription rates (beta-blockers plus an ACE-I/ARB/ARNI plus an MRA) did not significantly increase (56.1–57.7%).^120^ Such indicated the need for digital strategies to be transitional, ensuring continuity of care and adherence within the community setting. The SUPPORT-HF2 was a RCT that involved seven clinical sites in the UK and recruited a total of 202 patients with HFrEF.^121^ Patients randomized to the intervention received additional regular feedback via a telephone to support self-management, and their primary care clinicians received digital instructions on how to deal with results from recent blood investigations and implications for pharmacological treatment. The investigators found no improvement in GDMT prescription rate as a result of the intervention. In today’s current era, there are a growing number of smartphone applications to aid direct communication such as video consultation between clinicians and patients.

### Best practices and gaps in knowledge

Results from a few small experimental RCTs indicate that multifaceted digital interventions might improve clinical outcome. Uncertainties remain about which combinations of digital and non-digital interventions are most effective and adaptable across different healthcare settings. Best practices entail exploring the synergistic effects of these multifaceted interventions, aiming to enhance treatment adherence, reduce hospitalizations, and improve quality of life. Crucially, efforts should focus on ensuring these interventions complement rather than contradict each other, avoiding potential antagonistic effects that could undermine therapeutic benefits. Moving forward, quantifiable targets for advancing these technologies should include developing novel trial designs that accurately assess the combined impact of digital interventions on patient health outcomes. Establishing standards for interoperability and data integration across platforms is essential to facilitate seamless communication between different digital tools and healthcare systems. Moreover, defining measurable outcome metrics such as adherence rates and patient satisfaction scores will be critical for evaluating the effectiveness of multifaceted approaches. Standardizing these metrics will enable consistent evaluation and comparison of interventions, fostering evidence-based practices in HF management.

## Equal access in healthcare, subgroups, and patient preferences

Heart failure is a condition that affects individuals of all backgrounds, but there are well-documented disparities in its prevalence, management, and outcomes among minority populations.^122^ Health disparities contribute to the low GDMT prescriptions rates, with a reported 7.5% lower adherence among some minorities around the world.^123^ For example, fewer patients with HFrEF in low-income countries receive GDMT prescription than patients living in high-income countries. Socioeconomic factors, including insurance coverage and access to specialty care, can significantly impact the GDMT prescriptions rates. However, digital solutions may only be feasible if the patient has access to a suitable device and internet access.^80,124^ To prevent disparities, digital solutions should be made available and designed for groups that currently have limited use of digital technology, such as older people as well as those with lower educational attainment and income.^52,80^ Historically, elderly patients were reluctant to engage in digital solutions; however, more and more elderly patients own a smart device, therefore making its implementation into HF care easier. Frailty has been shown to interfere with GDMT prescription rate in patients with HFrEF.^125^ These patients are therefore at a higher risk of decompensation. Patients with HF with a less common aetiology such as cardio-oncology patients are demonstrated to benefit from optimization of GDMT prescription rate.^126–129^ The recent SMART-BREAST trial assessed the role of the BREASTMATE App to improve exercise activity in breast cancer patients receiving anthracycline chemotherapy and demonstrated a significant 46 m (interquartile range 28–63 m) improvement in six-minute walk test distance at follow-up after completing chemotherapy compared to controls.^130^ Patient preferences are important when considering digital solutions to optimize GDMT prescription rate in patients with HF. Knowledge about HF patients’ perceptions of technology use for self-care and a better understanding of issues associated with technology access and digital literacy skills can aid in the development and implementation of effective health behaviour interventions for clinicians, resulting in increased compliance, better outcomes, and lower healthcare costs.^131^

## Human barriers of digital solutions for optimization of guideline-directed medical therapy prescription rates

The implementation of digital solutions for optimization of GDMT prescriptions rates is a complex process.^74,132–134^ Addressing these implementation barriers requires a multidimensional approach involving collaboration among patients, clinicians, technology vendors, policymakers, and regulatory bodies.^135^ Clinician barriers to adopting digital solutions to change behaviour in HF care include scepticism about the effectiveness of these tools and resistance to altering established clinical practices. Additionally, the perceived complexity and time investment required for implementing digital solutions can deter their adoption. Investing in digital solutions for HF care involves not only initial implementation but also ongoing time and resources to ensure their effective use and integration into healthcare systems. Clinicians may initially experience an increased workload due to the need to interact with and interpret reports generated by digital solutions, which requires time for data analysis and patient communication. Additionally, the preferences of patients regarding the frequency and format of contact with their clinicians play a critical role as some patients with HF may prefer regular remote consultations, while others may opt for in-person visits. Insights from behavioural science further highlight those factors such as patient trust, perceived usefulness of the technology, ease of use, and personalized communication significantly influence the uptake of health advice. Therefore, a balanced approach that considers the practical workload of clinicians, patient preferences, and behavioural insights is essential for the successful adoption and sustained effectiveness of digital solutions in HF care. However, by addressing the challenges outlined, it is possible to overcome these barriers and achieve the benefits of digital solutions. The ESC is involved with digital solutions on many different levels. Its members deal with the changes in practice that Information and communications technology innovation brings, and dedicated digital health committees are instituted with activities such as position papers.

Patients with digital literacy experience barriers on application of digital solutions. The World Health Federation provided a roadmap for deployment of digital health in cardiology.^135^ Prospective validation is needed of the efficacy of this roadmap. Validation of digital solutions remains scattered. Concern of regulation and data training also has been raised worldwide, calling us to determine safety boundaries for best digital utilization.

## Technical, legal, and ethical aspects of digital solutions for optimization of guideline-directed medical therapy prescription rates

Technical barriers are one of the most common challenges, for example there may be a lack of interoperability between different EHR systems and digital solutions to optimize GDMT prescriptions rates. This can make it difficult to share patient data between different systems, which is essential for providing coordinated care. Additionally, digital solutions to optimize GDMT prescription rates may not have user-friendly interfaces that are easy to navigate and use. This can be a barrier for clinicians who are not familiar with technology.^136^

Furthermore, digital solutions involve the collection, storage, and transmission of sensitive patient data. Protecting patient privacy and maintaining data security is essential, but it can also adhere to local regulations that may delay data sharing. Informed consent from the patient is a necessity. There may be regulatory hurdles that need to be overcome before a digital solution to optimize GDMT prescription rates can be implemented.^136^ If digital solutions for HF care qualify as medical devices, they must adhere to stringent regulatory requirements to ensure their safety, efficacy, and reliability. These regulations are crucial for protecting patients and ensuring that the solutions provide tangible clinical benefits, underscoring the need for rigorous evaluation and oversight.^137^

Workflow integration can also represent a challenge. Healthcare providers often have established routines and processes, and introducing new technologies may disrupt their established ways of working. Training and education are key for a successful implementation, but it is not always easy to undertake due to staff and time constraints. Training programmes should address not only the technical aspects of using digital solutions but also focus on workflow integration, best practices, and ensuring that providers understand the clinical context and significance of GDMT prescription rates. With increased implementation of digital solutions, improved knowledge, and awareness for patients that frequently someone reviews the data can help establish trustworthy solutions. Healthcare providers, particularly those who are not technology savvy, may be resistant to adopting digital solutions. Culturally and linguistically diverse population can struggle to engage with digital health technologies.^138^ Finally, financial barriers need to be considered, despite cost-effective studies on digital solutions to optimize GDMT prescription rates lacking. However, optimization of pharmacotherapy in patients with HFrEF is highly cost effective and can result in cost savings.^139^

## Future directions

While modest to moderate increases in GDMT prescriptions have been observed during research evaluations of digital interventions, and not all studies have shown positive results, it cannot be assumed that these increases will translate into better outcomes. Most of these improvements have not yet been confirmed on clinical endpoints, highlighting the need for dedicated outcome studies with clinically relevant endpoints to assess the impact of GDMT prescriptions resulting from digital solutions in patients with HF. Looking to the future, experience and studies in the use of digital consults and digital remote monitoring should be encouraged, with clinicians actively alerted to patients on suboptimal GDMT. However, the digital solutions must balance the frequency of alerts to ensure effectiveness without causing clinicians to experience alert fatigue, which can result in important notifications being ignored. Proper design is crucial to maintain long-term engagement and compliance while not overwhelming the clinicians. Longitudinal data are required to verify that the benefits of digital solutions persist over time. Hawthorne bias in the studies discussed occurs when patients changed their behaviour because they know they were being observed. While a positive effect is expected during the study to optimize GDMT, its sustainability post-study is uncertain.

Researchers should also consider study designs that fit the unique sociotechnical aspects of digital solutions. These solutions include (i) qualitative research to understand the lived experiences and perspectives of patients with HF and clinicians using digital solutions; (ii) action research to engage stakeholders in iterative cycles of problem identification, solution development, and evaluation to co-create effective digital solutions; and (iii) case studies to deeply analyse specific instances of digital solution implementation to uncover contextual factors influencing success or challenges. For AI, DECIDE-AI recommendations are available.^140^ These designs provide nuanced insights into how technology interacts with social and organizational dynamics in healthcare settings.

## Conclusion

This Clinical Consensus Statement presents definitions and explanations of key concepts in digital solutions in GDMT in patients with HF, summarizes evidence from peer-reviewed papers, gives insights on clinical practice, and identifies gaps in evidence that require attention. Digital solutions are a heterogeneous term that encompasses remote monitoring, remote treatment, and remote conversation using a variety of technologies. To this date, there is limited evidence from large cohort studies or RCTs that supports the use of AI for GMDT. Although promising results from first (small-scale) randomized trials and observational cohort studies are acknowledged, this needs to be investigated. Addressing barriers necessitates a comprehensive approach involving collaboration among patients, healthcare providers, technology vendors, policymakers, and regulatory bodies.

## Data Availability

The data underlying this clinical consensus statement are derived from publicly available sources. All articles and data sets analysed are available in PubMed, Web of Science, and the Cochrane library and can be accessed through the references listed in the manuscript.
